# Climate Change Adaptation, Social Resilience, and Perceived Values Data from Turkana, Machakos and Narok Counties, Kenya

**DOI:** 10.1016/j.dib.2024.110978

**Published:** 2024-09-26

**Authors:** Beatrice Stockport, Pu Yang, James Kimani, Alycia Leonard, Stephanie Hirmer

**Affiliations:** a Department of Engineering Science, University of Oxford, Oxford OX1 3PJ, United Kingdom; b JapakGIS Limited, Garden Estate Road, Nairobi City, Kenya

**Keywords:** Energy planning, Infrastructure planning, Sustainable development, Socioeconomic status, LMICs, Community needs, Climate shocks, Drought

## Abstract

This dataset provides socioeconomic and value perception interview data collected from 1,021 individuals living across three counties in Kenya: Turkana, Machakos and Narok. The data are made available with sub-county level geospatial attribution. Socioeconomic data were collected on housing, healthcare, water sources, electricity access, experience of extreme weather events, community services, and access to information. Value perception data were collected using the user-perceived value (UPV) method – a perception-based surveying approach which requires interviewees to select their most valued household items in different circumstances, and explain their choice through ‘why’-probing. For this dataset, the UPV method was used to identify the most valued household items in daily life and in the event of an extreme weather event, e.g. a drought, floor or heatwave. Together, the socioeconomic and interview data can be used to better understand the views of different communities and demographic groups across Kenya concerning climate change and extreme weather events. They also provide insight about the intrinsic, social, emotional, epistemic, functional, and indigenous value associated with everyday household items. The data can be used by policymakers to inform development planning and to identify gaps in the available infrastructure. Additionally, researchers and development practitioners can use these data to design interventions which reflect the needs and values of communities in Kenya.

Specifications Table

**Table:** 

Subject	Social Science
Specific subject area	Planning and Development
Type of data	Raw, Analysed
Data collection	Data were collected through face-to-face interviews, which included qualitative and quantitative socioeconomic survey questions, and open-ended perceived-value questions. The data collection process involved targeted sampling within communities so that the dataset has approximately equal gender representation, and includes individuals of different ages, occupations, levels of income, and levels of education. Data collection coordinators within each county recruited participants from a variety of venues, including restaurants, places of worship, markets and community organisations, with the aim of increasing sampling diversity. Consent was obtained from every participant.
Data source location	Turkana, Machakos and Narok Counties, Kenya
Data accessibility	Repository name: Zenodo Data identification number: 10.5281/zenodo.11242112 Direct URL to data: https://doi.org/10.5281/zenodo.11242112

## 1 Value of the Data

• This dataset is important to understand the socioeconomic status, perceptions and values of individuals in rural Kenya, and to provide information on their access to community services, energy and water as they relate to climate change preparedness.
• These data can be utilised to evaluate resilience, exposure and vulnerability to extreme weather events at an individual, household, sub-county or county level. This analysis can be used to inform infrastructure, service and development planning.
• The data on access to community services, energy and water can be used alongside the sub-county geospatial tagging to identify the state of existing infrastructure. This analysis can be used to inform infrastructure planning.
• The data on perceptions of extreme weather events and environmental hazards, degree of preparation for such hazards, the ability to respond and recover, and access to information can be used by researchers and development practitioners to conduct disaster risk analysis.
• The verbatim interview text can be used by policymakers and non-governmental organisations (NGOs) to understand individuals’ perceptions of climate change and design social awareness campaigns.
• If this data collection were repeated, these data could contribute towards a time series dataset. The time series dataset could be used to evaluate the effect of energy access, climate change adaptation and development programmes on poverty levels, resilience to extreme weather events and quality of life.

## 2 Background

Extreme weather events, such as droughts, floods and heatwaves, are projected to increase in frequency and severity as a result of climate change [1]. Kenya is already experiencing the impacts of extreme weather events, including failing crops, damaged infrastructure and housing, reduced water availability, and heat- and disaster-related mortality [2].

To develop effective strategies for reducing poverty and improving living standards in the context of increasing extreme weather events, infrastructure and services must be designed to meet communities’ needs and build resilience. Resilience is defined here as the ability to anticipate, absorb, adapt and recover from the impact of extreme weather events. Additionally, development initiatives should be designed to reflect individuals’ values to improve user acceptance, and reduce the risk of stranded assets [3]. This is especially important for vulnerable individuals or communities, who are more susceptible to extreme weather events by virtue of conditions determined by physical, social, economic or environmental factors [1]. Increasing energy access across Kenya, particularly via renewable energy, can improve living standards, enhance resilience, and enable climate change adaptation [4].

The objective of this data collection was to understand how energy services can be designed to build resilience to extreme weather events and enable climate change adaptation, while aligning with community values. These data were also gathered to better understand the needs of rural Kenyan communities and the value associated with particular household items or appliances (e.g., a grain mill, jerrycan or generator). Presently, these data are being applied to inform the ongoing county-level energy planning process in Kenya, given the recent requirement for every county to produce a county energy plan [5]. However, the limitations of this dataset must be considered if it is being used to inform spending on energy services and infrastructure.

## 3 Data Description

The dataset presents socioeconomic survey and interview data collected from 1,021 participants in Turkana, Machakos and Narok Counties, Kenya. This dataset can be used to understand how county-level energy planning could account for the forecasted energy demand of resilience-building initiatives, climate adaptation measures, and improving living standards through equitable development initiatives. These data add to those already published from Siaya County, Kenya [7].

• **Turkana** has a population of 926,976, of which 48.4 % (448,868) are female and 84.8 % (786,185) live in rural areas [6]. There are a total of 164,519 households with an average size of 5.63 [6]. Turkana County is mainly arid with unreliable rainfall [8]. Given the issue of water scarcity, the economy of Turkana is primarily based on pastoral farming and fishing [8]. There is also a lack of basic infrastructure across the county, including roads, markets, health centres and schools, and poverty levels in Turkana are high [8].
• **Machakos** has a population of 1,421,932, of which 50.0 % (711,191) are female and 70.8 % (1,007,854) live in rural areas [6]. There are a total of 402,466 households with an average size of 3.53 [6]. Machakos County has moderate and seasonal rainfall, and a varied landscape [9]. Agriculture, pastoral farming and businesses are the main economic activities across the county, and it has also been chosen as the location of a new technology hub, *Konza Technopolis* [10,11]. Machakos has undergone significant development in recent years, including improved road networks, healthcare facilities, schools and water infrastructure [9].
• **Narok** has a population of 1,157,873, of which 50.0 % (578,805) are female and 91.3 % (1,007,854) live in rural areas [6]. There are a total of 241,125 households with an average size of 4.80 [6]. The climate in Narok varies from semi-arid to semi-humid, with two rainy seasons [12]. The economy is primarily based on agriculture, pastoral farming, and tourism [12]. Narok is in various stages of development, with rural areas having limited access to education and healthcare [12]. However, the County Government is implementing multiple development initiatives, such as commissioning a modern abattoir, building a road in Olokurto ward and launching a bursary programme [12].

The dataset is separated into two parts: (1) household characteristics, socioeconomic data and demographics; and (2) verbatim personal value perceptions, gathered through UPV interviews with community members. Given the different states of development, economic activities and agroclimatic conditions between Turkana, Machakos and Narok, these data provide insights into the variety of challenges faced by diverse communities across Kenya. These can include poverty, lack of infrastructure, extreme weather events, difficulty accessing community services, poor access to information, unemployment, or having a disability.

**Fig. 1:**
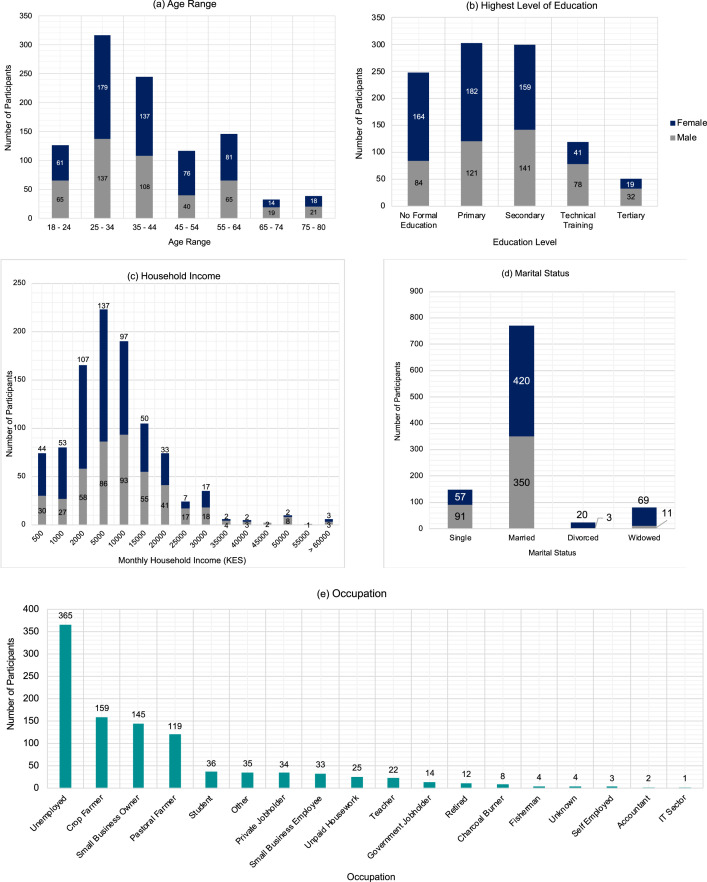
Demographic characteristics of survey participants, showing; (a) age range, (b) highest level of education, (c) monthly household income, (d) marital status, and (e) occupation.

Fig. 1 illustrates the demographic characteristics of the 1,021 participants, obtained from the socioeconomic survey. The sample consists of 455 males (44.6 %) and 566 females (55.4 %), with an age range of 18–80 (Fig. 1a). The majority of participants were married (75.4 %), 14.5 % were single, 7.8 % were widowed and 2.3 % were divorced (Fig. 1d). In the dataset, 2.7 % participants reported having a disability.

In this sample, more females than males have not received a formal education or have only received primary or secondary education, whereas more males than females have gone on to receive technical training or attend university (Fig. 1b). In total, 24.3 % of participants have received no formal education, 30.0 % have received primary education, 29.4 % have received secondary education, 11.7 % have received technical training, i.e. gone to college, and 5.0 % have attended university (Fig. 1b). The monthly household income of participants ranged from 500 Kenyan Shilling (KES) to over 60,000 KES (Fig. 1c). The largest occupational group represented in the sample are the unemployed (35.7 %), followed by crop farmers (15.6 %), small business owners (14.2 %), and pastoral farmers (11.7 %) (Fig. 1e). Of employed survey participants, 48.4 % work in agriculture, 46.8 % in services and 1.9 % in industry, with 6.7 % unknown. Comparing to national statistics, 53.8 % of those employed in Kenya work in agriculture, 38.7 % in services, and 7.4 % in industry [13]. In the survey sample, 42.9 % of participants are unemployed or outside the labour force, by virtue of being full-time students, home makers, retired, elderly, too young, or living with a disability. This is less than the national level in Kenya, where 54.3 % of the population are unemployed or outside the labour force [14].

### 3.1 Socioeconomic data

The socioeconomic data is presented in *Kenya_UPV_Survey.csv*. The column headings represent each survey question and each interviewee's response to the questions are recorded in individual rows. A unique, anonymised interview ID is provided for each interviewee which can be used to cross-reference their responses in *Kenya_UPV_Utterances.csv*.

These data provide information on demographics (column 13–20), household information (21–36), inclusion (37–39), housing (40–44), healthcare (47–51), water sources (52–64), energy access (65–78), experience of extreme weather events (79–112), community services (113–123), and access to information (124–140).

### 3.2 Value perception data

**Fig. 2:**
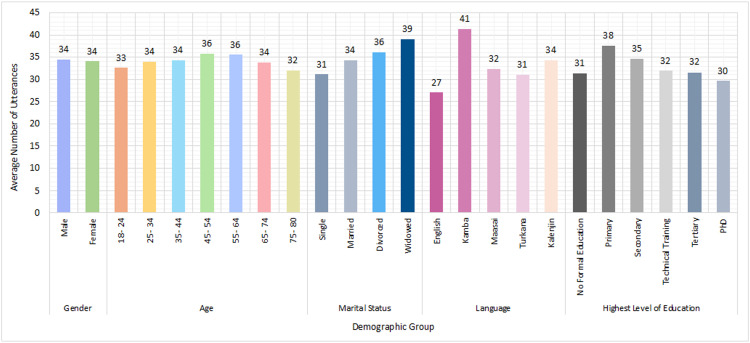
Average number of utterances for different demographic groups: (a) gender, (b) age, (c) marital status, (d) language, and (e) level of education.

The value perception data are presented in *Kenya_UPV_Utterances.csv*. This file includes one record (i.e., row) per utterance (i.e., sentence) stated by the interviewee during why-probing on the importance of their selected items. There are 34,281 rows in total. Fig. 2 shows the average number of utterances for different demographic groups. The *Kenya_UPV_Utterances.csv* file also includes the sentiment and value associated with each utterance, as well as the interviewee's unique ID to cross-reference their responses to the socioeconomic survey data in *Kenya_UPV_Survey.csv.*

The data in *Kenya_UPV_Utterances.csv* provide information on:

1. The five household items that interviewees perceive as most important in daily life.
2. The three household items that interviewees perceive as the most important in the event of an extreme weather event.
3. The energy-enabled appliances that interviewees perceive to be the most and least useful.

### 3.3 Codebook

The file *upv_definitions.py* is a codebook that describes the values that were used to annotate the interviewees’ responses.

### 3.4 Questionnaire

The file *Questionnaire_Kenya_Dataset.pdf* is a copy of the questionnaire used for data collection.

## 4 Experimental Design, Materials and Methods

### 4.1 Study preparation

To ensure the data collection was ethical, a risk and ethics assessment for research involving human participants was undertaken and approved by the National Commission for Science, Technology and Innovation (NACOSTI) ethics board, under license number NACOSTI/P/23/21652, and the Medical Sciences Interdivisional Research Ethics Committee (MS IDREC) at the University of Oxford, under reference: R83092/RE001.

### 4.2 Site selection

Kenya was selected as the location for this data collection because every county has recently been mandated to produce a county energy plan [5]. This data collection was therefore undertaken to understand how county-level energy planning could account for the forecasted energy demand of resilience-building initiatives, climate adaptation measures and improving living standards through equitable development initiatives.

Turkana, Machakos and Narok Counties were selected as the locations for data collection given their varying levels of development, infrastructure, economic opportunities, and agro-climatic conditions. This dataset therefore presents a diverse range of perspectives and can be used to compare experiences, perceptions, and values of communities across Kenya.

### 4.3 Sampling strategy

The participant selection was guided by the project coordinator and facilitated by the Turkana, Machakos and Narok County Commissioner's Offices. Over a period of three weeks, 316 individuals from Turkana, 399 individuals from Machakos and 288 individuals from Narok were interviewed.

To ensure representation of the diverse population, a targeted community sampling approach was used to identify participants. In total, data was collected from twenty villages out of the thirty in Turkana and Narok, respectively, and twenty villages out of the forty in Machakos. Participants were purposefully selected to provide approximately equal participation of males and females, as well as representing community members with a range of ages, occupations, levels of education, and people living with disabilities.

### 4.4 Data collection

The socioeconomic survey and interviews were conducted face-to-face. The data was recorded on a tablet using KoBo Toolbox - a digital mobile application.

During the interviews, the UPV method was used to identify and understand the value associated with particular items. This method is derived from [3].

In the UPV method, the items are graphically depicted for interpretability and accessibility, for example, if a participant is illiterate. Participants were asked to select the five everyday items that they value most from a set of forty-five that are found in the case study communities. The full list of possible items is provided in *Questionnaire_Kenya_Dataset.pdf.* Out of the forty-five items, eight are energy appliances. They are a fan, refrigerator, television, water pump, motorbike, pressure cooker, solar photovoltaic (PV) system and grain mill. Subsequently, the participants were asked for the reasons behind their selections in three-rounds of 'why'-probing, i.e., questioning, as described in [15] and [3]. Additionally, three open-ended questions were asked on participants' attitudes and perceptions towards climate change.

The interviews were either conducted in English or the local language: Maasai, Kalenjin (Narok), Kamba (Machakos) and Turkana (Turkana). As per the recommendation of local organisations and considering local norms, interviewees were recompensed for their participation [15]**.**

### 4.5 Data processing

The interview transcripts were translated into English by local translators. The interviews were then separated into paragraphs and utterances, which were tagged with the sentiment and associated value using natural language processing (NLP), as described in [16]. For example, the value associated with boreholes is *water security*. The interview and socioeconomic survey data were collated in Microsoft Excel. To protect the interviewees’ identities, all names and means of identification have been removed from the dataset, thereby conforming with the NACOSTI ethical procedure. A summary of the data collection process is provided in Table 1. The data collection, translation and annotation has been managed by *JapakGIS*; a data collection company based in Kenya.

**Table 1:** Summary of data collection process.

**Data Collection**	Timeline	2023
	Location	Turkana, Machakos and Narok Counties, Kenya
	Collectors	Interviews were conducted by Kenyans fluent in the local language who had been trained on data collection in a workshop.
	Methodology	The interviews were held at the interviewees’ homes. The UPV method was conducted in a semi-structured manner to avoid direct inquiry from the interviewer. Additionally, each interviewee completed the socioeconomic survey.
**Data Translation**	Timeline	2023
	Location	Online
	Translators	The translators were Kenyan citizens who spoke the interviewees’ language and English fluently.
**Data Annotation**	Timeline	2023
	Location	Online
	Annotators	Trained and experienced annotators, primarily from Kenya, undertook the annotation.
	Methodology	Each utterance was annotated with the sentiment and associated value. To ensure reliability, each sentence was separately annotated by five different annotators. Only labels where a minimum of three of the five annotators agreed were used. This was to ensure consistency and quality of the annotations.

## Limitations

The data was collected in different languages depending on the location of the interviewee. Bias could therefore have been introduced into the dataset upon translation of the interviews into English, due to differences in vocabulary between languages. This has been partially mitigated by having multiple translators and annotators for each data entry, and ensuring that the translation and annotation align.

There are also some data entries that are missing data points because of inaudible interview recordings. To maintain the quality of the dataset, twenty-five data entries with multiple missing data points have been removed. If this dataset will be used to inform spending on energy services, infrastructure or development initiatives, careful assessment must be made about whether this dataset is nationally representative.

Furthermore, interviewees were recompensed for their participation after consultation with local organisations, and consideration of local norms and precedents set by previous researchers. However, renumeration is ethically-charged and widely contested because it can lead people to participate in data collection against their judgement, introducing sampling bias or creating power dynamics resulting in acquiescence bias [15].

## Ethics Statement

To ensure the study's integrity, a risk and ethics assessment was undertaken and evaluated by the Medical Sciences Interdivisional Research Ethics Committee (MS IDREC) at the University of Oxford in accordance with the procedures outlined for all research involving human participants. It was completed and approved with reference: R83092/RE001. Additionally, a risk and ethics assessment was completed for the NACOSTI ethics board. This was approved under license number NACOSTI/P/23/21652 for research involving human participants.

Informed consent was obtained from all interviewees and all identifying information has been removed to protect interviewees' identity.

## CRediT authorship contribution statement

**Beatrice Stockport:** Data curation, Writing – original draft, Visualization, Writing – review & editing. **Pu Yang:** Data curation, Writing – review & editing, Supervision. **James Kimani:** Investigation, Data curation. **Alycia Leonard:** Writing – review & editing, Supervision. **Stephanie Hirmer:** Funding acquisition, Conceptualization, Validation, Writing – review & editing, Supervision.

## Data Availability

Climate Change Adaptation, Social Resilience, and Perceived Values Data from Turkana, Machakos and Narok County, Kenya (Original data) (Zenodo).
